# Time‐based measures for quantifying disease modification in Alzheimer’s disease

**DOI:** 10.1002/alz.087702

**Published:** 2025-01-09

**Authors:** Lars Lau Raket

**Affiliations:** ^1^ Eli Lilly and Company, Indianapolis, IN USA

## Abstract

**Background:**

The advent of disease‐modifying therapies in Alzheimer’s disease (AD) necessitates a nuanced understanding of how therapies impact disease processes. Over the past decades, AD clinical trials have primarily relied on classical statistical analysis methodology such as the mixed model for repeated measures (MMRM) to estimate treatment effects. These conventional treatment effect quantifications are given as group differences in clinical outcome measures at a single visit. While this classical approach of estimating treatment effects is well established, the resulting quantifications have shortcomings in relation to data utilization, meaningfulness, cumulative benefit summarization, post‐trial implications, and cross‐trial comparability.

**Method:**

Properties of conventional treatment effect quantifications from the MMRM were compared with two time‐based quantifications from the progression model for repeated measures (PMRM)^1^ and a latent time‐disease progression model. Results were illustrated and compared using data from the TRAILBLAZER‐ALZ 2 trial of donanemab.

**Result:**

The MMRM had fewest assumptions, followed by PMRM and then the laten‐time quantifications. PMRM and latent‐time quantifications utilized information across visits better than the conventional MMRM quantification and produced greater power to detect treatment effects. Compared to conventional quantifications, the time‐based quantifications of treatment effects offered several desirable properties in terms of meaningfulness, cumulative benefit summarization, post‐trial implications, and cross‐trial comparability.

**Conclusion:**

Time‐based estimates, particularly those derived from PMRM and latent time disease progression models, offer a set of desirable properties that complement conventional treatment effect quantifications. This study advocates for the inclusion of time‐based measures in the evaluation of disease modification in AD, providing a more comprehensive and nuanced perspective for guiding future clinical trial methodologies and result interpretations.

**References**:

1. Raket, L. L. (2022). Progression models for repeated measures: Estimating novel treatment effects in progressive diseases. *Statistics in Medicine*, *41*(28), 5537‐5557.

